# Estrogen receptors and extracellular matrix: the critical interplay in cancer development and progression

**DOI:** 10.1111/febs.17270

**Published:** 2024-09-16

**Authors:** Sylvia Mangani, Zoi Piperigkou, Nikolaos E. Koletsis, Paraskevi Ioannou, Nikos K. Karamanos

**Affiliations:** ^1^ Biochemistry, Biochemical Analysis & Matrix Pathobiology Research Group, Laboratory of Biochemistry, Department of Chemistry University of Patras Greece

**Keywords:** breast cancer, cell signaling, epigenetics, estrogen receptor alpha, estrogen receptor beta, extracellular matrix, molecular targeting

## Abstract

Cancer remains a significant global health concern. Breast cancer is a multifaceted and prevalent disease influenced by several factors, among which estrogen receptors (ERs) and the extracellular matrix (ECM) play pivotal roles. ERs, encompassing ERα and ERβ, exert significant diversity on tumor behavior, cell signaling, invasion, and metastatic potential, thus guiding breast cancer prognosis. Understanding the multifunctional connections between ERs and ECM that mediate the dynamics of tumor microenvironment is vital for unraveling the complexity of breast cancer pathobiology and identifying novel therapeutic targets. This critical review delves into the intricate nature of ERs, emphasizing their structural isoforms and the consequential impact on breast cancer outcomes. A detailed examination of ER‐mediated cell signaling pathways reveals how differential expression of ERα and ERβ isoforms influence breast cancer cell behavior. The functional ERs‐matrix interactions emerge as a pivotal factor in modulating epigenetic mechanisms of breast cancer cells, orchestrating changes in cellular phenotype and expression patterns of matrix modulators. Specifically, ERα isoforms are shown to regulate ECM signaling cascades, while the effects of ECM components on ERα activity highlight a bidirectional regulatory axis. The diversity of ERβ isoforms is also highlighted, illustrating their distinct contribution to ECM‐mediated cellular responses. This review underscores the complex interplay between ERα/β isoforms and the ECM, shedding light onto the potential therapeutic strategies targeting these interactions to improve breast cancer management.

## Graphical abstract

Breast cancer is a complex disease significantly influenced by estrogen receptors (ERs) and the extracellular matrix (ECM), both of which are critical in shaping tumor behavior. ERα and ERβ modulate cancer progression and therapeutic response with their interactions intricately linked to ECM dynamics. The ECM, integral to the tumor microenvironment, cooperates with ERs to regulate cellular functions. Deciphering the ERs‐ECM complex interactions offers the potential for advancing breast cancer therapies.

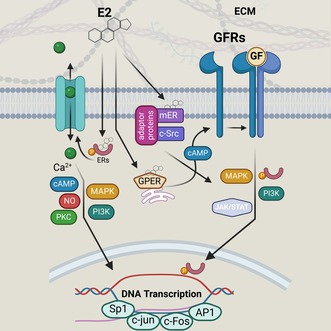

**Abbreviations**
ADAM22: disintegrin and metalloproteinase domain‐containing protein 22
AF: activation function domains
AKT: protein kinase B
AP1: activator protein 1
CAFs: cancer‐associated fibroblasts
cAMP: cyclic adenosine monophosphate
CCN1: cellular communication network factor 1
CD44: cluster of differentiation 44
CDH1: E‐cadherin gene
COL1A1: collagen type I alpha 1 chain
COL4A1: collagen type IV alpha 1 chain
CSCs: cancer stem cells
c‐Src: cellular Src kinase
CXCR4: CXC motif chemokine receptor 4
DBD: DNA‐binding domain
E2: estradiol
ECM: extracellular matrix
EGFR: epidermal growth factor receptor
EMT: epithelial‐to‐mesenchymal transition
ER: estrogen receptor
ERE: estrogen response element
ERINA: estrogen‐inducible lncRNA
Erk1/2: extracellular signal‐regulated kinase 1/2
ERα/ESR1: estrogen receptor alpha/estrogen receptor alpha gene
ERβ/ESR2: estrogen receptor beta/estrogen receptor beta gene
EVs: extracellular vesicles
FGF2: fibroblast growth factor 2
GFs/GFRs: growth factors/growth factor receptors
GPER: G‐protein coupled estrogen receptor
HD: hinge domain
HER2: human epidermal growth factor receptor 2
HOTAIR: HOX transcript antisense RNA
HPSE: heparinase
HSPG: heparan sulfate proteoglycan
ICAM‐1: intracellular adhesion molecule 1
IGF‐IR/IGF1R: insulin‐like growth factor 1 receptor/insulin‐like growth factor 1 receptor gene
IL: interleukin
IR: insulin receptor
JNK: Janus kinase
LBD: ligand‐binding domain
lncRNA: long non‐coding RNA
LOX/LOXLs: lysyl oxidases/LOX‐like enzymes
MALAT1: metastasis‐associated lung adenocarcinoma transcript 1
MAPK: mitogen‐activated protein kinase
mER: membrane estrogen receptor
MICAL‐L2: molecules interacting with CasL (MICAL)‐like protein 2
miRNA: microRNA
MMPs: matrix metalloproteinases
mTOR: mammalian target of rapamycin
NO: nitric oxide
NTD: N‐terminal domain
ORF: open reading frame
PAI‐1: plasminogen activator inhibitor 1
PI3K: phosphoinositide 3‐kinase
PKA: protein kinase A
PKC: protein kinase C
PMN: pre‐metastatic niche
PR: progesterone receptor
PRLR: prolactin receptor
SDC: syndecan
SERMs: selective estrogen receptor modulators
SERPINE1: serpin family E member 1
Slug: zinc finger transcription factor
Snail1: snail family transcriptional repressor 1
Sp1: specificity protein 1
STATs: signal transducers and activators of transcription
TGF‐β: transforming growth factor beta
TIMPs: tissue inhibitors of metalloproteinases
TME: tumor microenvironment
TMPO‐AS1: thymopoietin antisense transcript 1
TNBC: triple‐negative breast cancer
tPA: tissue‐type plasminogen activator
uPA: urokinase‐type plasminogen activator
UTR: untranslated region
VEGF: vascular endothelial growth factor
VIM: vimentin
ZEB1: zinc finger E‐box binding homeobox 1

## Introduction

In recent years, breast cancer remains a significant global health concern, with statistics reflecting its prevalence and impact. According to the World Health Organization (WHO), breast cancer is the most commonly diagnosed cancer among women worldwide, with approximately 2.3 million new cases reported annually [1]. Moreover, it is the leading cause of cancer‐related deaths in women, accounting for over 600 000 deaths each year. While mortality rates vary across regions, advancements in early detection, diagnosis, and treatment have contributed to improved survival rates in many parts of the world.

Estrogen receptor alpha (ERα) and beta (ERβ) are involved in breast cancer development and progression. ERα, primarily expressed in the mammary epithelium, promotes cell proliferation in response to estrogen stimulation, making it a crucial therapeutic target in hormone receptor‐positive breast cancer. On the other hand, ERβ, with its diverse expression patterns and distinct signaling pathways, exerts both overlapping and opposing effects to ERα, influencing tumor growth, metastasis, and response to endocrine therapy [2]. Understanding the interplay between these receptors is essential for developing more effective targeted therapies and improving patient outcomes in breast cancer management. ERα‐positive (ERα+) breast cancer constitutes a substantial portion of diagnosed cases, comprising *ca* 70% of all breast cancer subtypes [3]. The understanding of the ER biology and the implications of ERs in breast cancer initiation, progress, and metastasis as well as disease treatment and management are emerging research fields.

Extracellular matrix (ECM), also known as intercellular matrix, constitutes a diverse array of biomolecules that provide structural support and signaling cues to cells in their surrounding microenvironment. ECM components include, among several macromolecules, collagens, fibronectin, elastin, laminin, proteoglycans/glycosaminoglycans, proteolytic and glycolytic enzymes, and matricellular proteins [4].

The tumor microenvironment (TME) refers to the cellular environment surrounding a tumor, consisting of blood vessels, immune cells, fibroblasts, and ECM components. The importance of the TME lies in its role in regulating various aspects of tumor growth, invasion, and metastasis. ECM molecules within the TME can influence cancer cell behavior by providing biochemical and biomechanical cues, as well as structural support, thus promoting cell functional properties, such as adhesion, proliferation, migration, and differentiation. Furthermore, cancer cell–ECM interactions modulate signaling pathways and facilitate interactions with immune cells. Understanding the dynamic interplay between tumor cells and the ECM within the TME is crucial for developing effective cancer therapies [5, 6, 7].

The interplay between estrogen/ERs signaling and matrix components is a key factor in ECM remodeling and, consequently, the regulation of various cell functional properties [2]. Recent studies have revealed a significant difference between the ECM derived from the normal mammary gland and the distinct subtypes of breast cancer. ECM remodeling in TME, the creation of the provisional matrix and the pre‐metastatic niche (PMN) are all critical steps that facilitate cancer progression and metastasis [8].

The main goal of this critical review is to explore the intricate interplay between the ER types and their isoforms with the ECM in driving breast cancer development, promotion, and metastasis. Insights into clinical implications and therapeutic opportunities targeting ERs–ECM dynamics are also presented and critically discussed.

## ERs structural diversity, expression in breast cancer prognosis and cell signaling

**Fig. 1:**
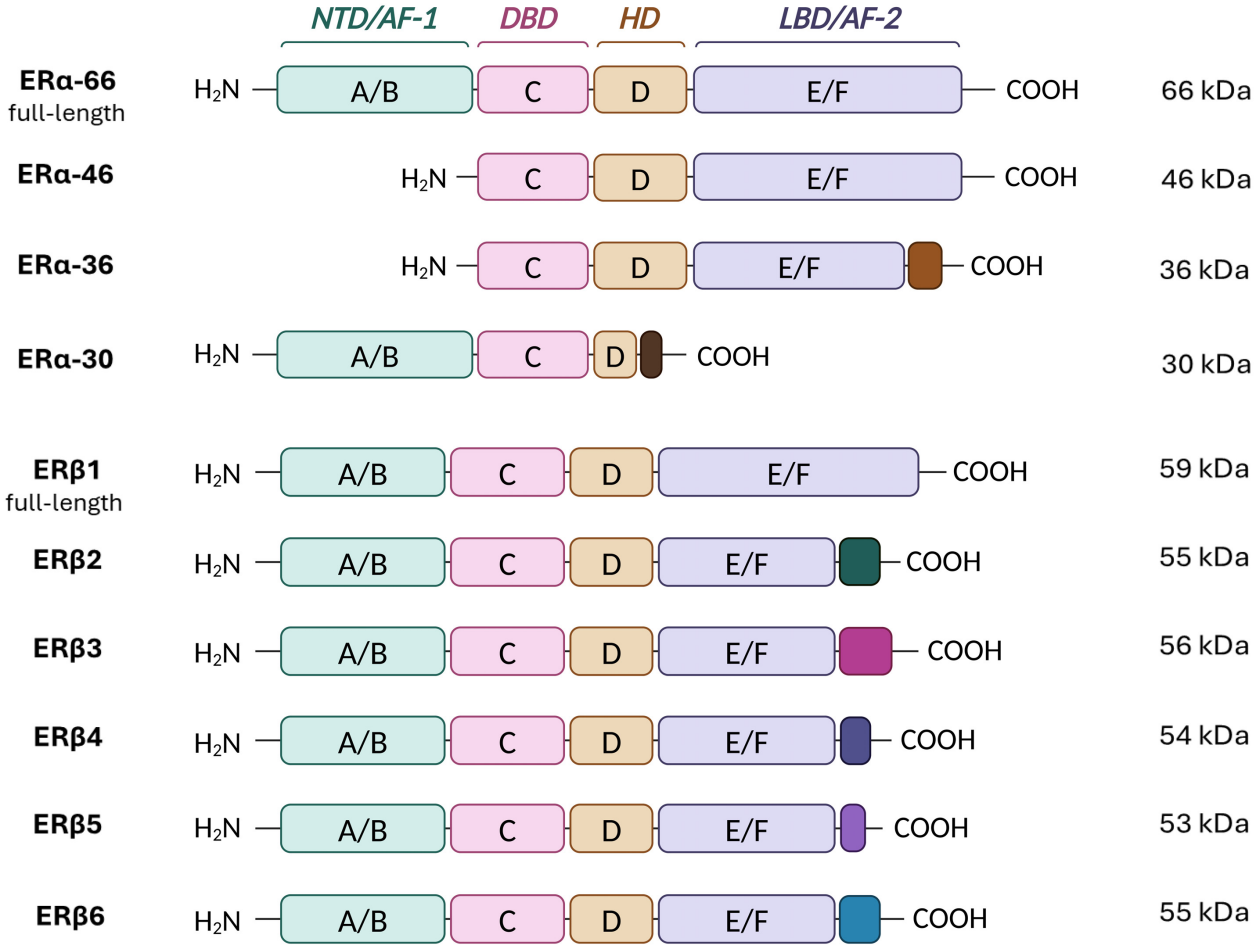
Schematic representation of the structural and functional domains of the major human estrogen receptors, ERα and ERβ. The main ERα isoforms include the full‐length protein, ERα‐66, along with shorter variants derived from alternative splicing of *ESR1* mRNA, ERα‐46, ‐36, and ‐30. The main ERβ isoforms, resulting from alternative splicing of *ESR2* mRNA, include ERβ1‐6. Distinct structural domains of ERα and ERβ isoforms are labeled A–F. Additionally, colored boxes observed in ERα‐36, ERα‐30, and ERβ2‐5 suggest the presence of distinct amino acid sequences at the C‐terminal end of each isoform. Created with BioRender.com. AF, activation function domains; DBD, DNA‐binding domain; ER, estrogen receptor; ESR1, estrogen receptor alpha gene; ESR2, estrogen receptor beta gene; HD, hinge domain; LBD, ligand‐binding domain; NTD, N‐terminal domain.

ERα and ERβ are members of the nuclear receptor superfamily for steroid/thyroid hormones, encoded by the *ESR1* and *ESR2* genes, respectively. Although they share a similar primary structure, crystal structure analyses have revealed only 47% sequence homology. Structurally, ERs consist of six modular domains (A–F), with varying levels of conservation. The N‐terminal A/B domain (NTD) contains the AF‐1 domain, which is essential for hormone‐independent transactivation, and exhibits the highest variability between ER subtypes. ER‐mediated gene transcription involves both AF‐1 and AF‐2 (NTD and LBD domains, respectively) activation, interacting with co‐regulatory proteins, such as Src. The highly conserved C domain contains the DNA‐binding domain (DBD), which is responsible for the dimerization of the receptor and the ER binding to estrogen response elements (EREs). Ligand‐induced conformational changes modulate ER transcriptional actions. Due to genetic and structural differences (Fig. 1), ERα and ERβ exert distinct functions in cancer progression [2].

**Fig. 2:**
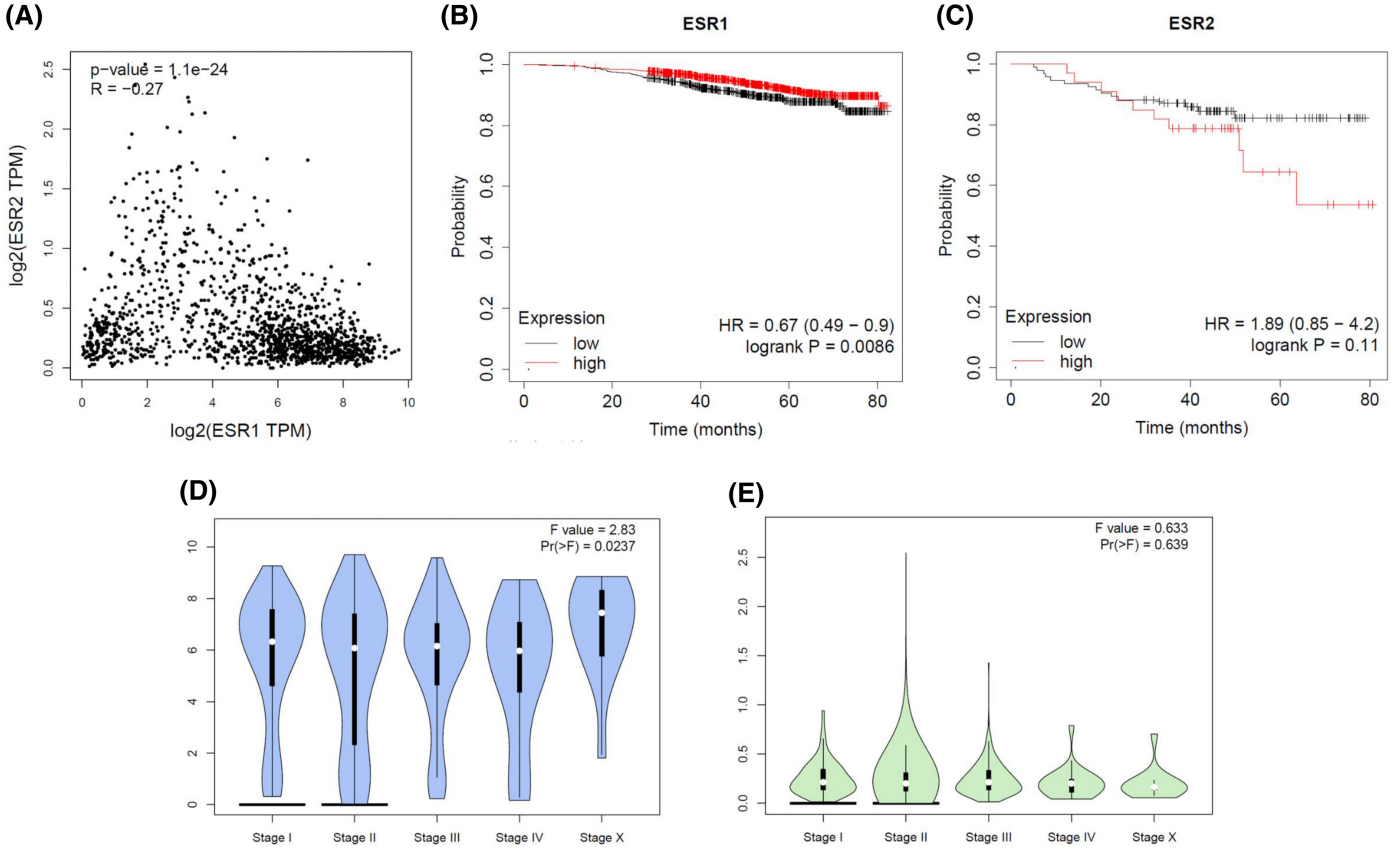
The prognostic potential of *ESR1* and *ESR2* in breast cancer. (A) Graphical representation of the correlation analysis between the *ESR1* and *ESR2* gene expression in both normal and tumor breast cancer. The correlation analysis tool GEPIA2 was utilized using the non‐log scale for calculation and the log‐scale axis for visualization and provides pairwise gene correlation analysis for given sets of TCGA Tumor and Cancer and GTEx expression data [81]. (B, C) Kaplan–Meier RNA‐seq survival analysis of the overall survival probability in low and high *ESR1* and *ESR2* expression, respectively, in TNBC. *P* value and hazard ratio (HR) value were calculated using a log‐rank test [82]. *ESR1* and *ESR2* refer to ERα and ERβ genes, respectively. (D, E) Stage plot showing the correlation between ESRs expression with tumor stage in breast cancer (GEPIA 2) [81]. ER, estrogen receptor; ESR1, estrogen receptor alpha gene; ESR2, estrogen receptor beta gene; TNBC, triple‐negative breast cancer.

*ESR1* and *ESR2* exhibit diverse alternatively spliced mRNA variants, with ERα having four and ERβ six variants, respectively (Fig. 1). Specifically, ERα includes shorter variants alongside the full‐length ERα‐66 protein, referred to as ERα‐46, ERα‐36, and ERα‐30 [9, 10, 11]. ERα‐46 exhibits a diverse range of expression levels across various types of human breast malignancies, occasionally even exceeding the abundance of the ERα‐66 protein, while ERα‐36 can antagonize ERα‐66 action [12, 13, 14]. ERα‐30, however, is not thoroughly described and may inhibit the expression of ERα‐66, functioning as a negative regulation variant [11]. ERβ, on the other side, exists in six different isoforms, ERβ1‐6, with ERβ1 comprising the full‐length isoform (Fig. 1). While ERβ1 acts mostly as an oncorepressor, ERβ2, ERβ4 and ERβ5 have been shown to promote breast cancer progression. For the roles of ERβ3 and ERβ6, limited data are available [15]. This diversity suggests different roles for ERα and ERβ, which are thought to act in opposing directions. This is also shown by the fact that the density of points in correlation analysis is shifted to high values for the *ESR1* and to low values for *ESR2*, respectively (Fig. 2A).

Meta‐analysis of retrospective data from breast cancer patients revealed that high levels of *ESR1* indicate better prognosis throughout an 80‐month timeframe in Luminal A subtype, compared to low *ESR1* expression (Fig. 2B). On the contrary, Kaplan–Meier survival analysis revealed that high levels of *ESR2* in triple‐negative breast cancer (TNBC) patients demonstrate a significantly lower overall survival, as compared to cases with low *ESR2* expression (Fig. 2C). This suggests the crucial role of ERβ in prognosis of aggressive breast cancer and that its targeting may be beneficial for effective management of this malignancy. Stage plot demonstrating the distribution of breast cancer stages within the study population shows that *ESR1* expression is relatively high throughout the malignancy progression (Fig. 2D), whereas *ESR2* expression shows no significant association with stage distribution (Fig. 2E).

**Fig. 3:**
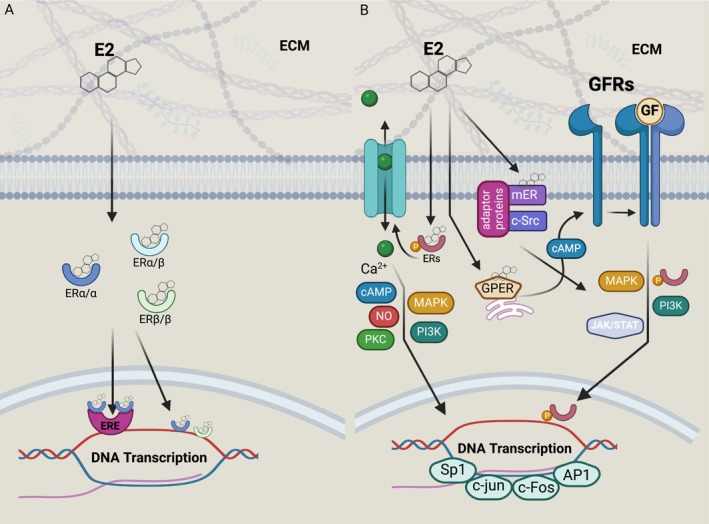
Mechanisms of E2‐activated signaling pathways. (A) The classical genomic pathway, through which the activated ER homo‐ and heterodimers regulate target gene expression by interacting with transcription factors. The activated ER complex translocates to the nucleus and binds to DNA, in an ERE‐dependent or ERE‐independent manner. ER binding to the DNA results in the recruitment of coactivators/corepressors, manipulating the transcription of target genes that mediate cellular functions including differentiation, proliferation, survival, etc. (B) In the non‐genomic actions, E2 binds either to GPER or to membrane‐associated ERs. E2 can also induce rapid changes in the intracellular Ca^2+^ levels, further mediating the subsequent cAMP/PKA pathway. E2 or phosphorylated ERs may also activate second messenger rapid signaling cascades, evoked by the RTKs for the activation of protein kinase signaling pathways (i.e. PI3K/AKT, MAPK, JAK/STAT), the phosphorylation of target proteins, thus activating several transcription factors, influencing critical cellular functions. Created with BioRender.com. AKT, protein kinase B; AP1, activator protein 1; cAMP, cyclic adenosine monophosphate; c‐Src, cellular Src kinase; E2, estradiol; ECM, extracellular matrix; ER, estrogen receptor; ERE, estrogen response elements; GF, growth factor; GFR, growth factor receptor; GPER, G‐protein coupled estrogen receptor; JAK, Janus kinase; MAPK, mitogen‐activated protein kinase; mER, membrane estrogen receptor; NO, nitric oxide; PI3K, phosphoinositide 3‐kinase; PKA, protein kinase A; PKC, protein kinase C; Sp1, specificity protein 1; STAT, signal transducers and activators of transcription.

Εstradiol (E2), the primary ligand for ERα and ERβ, constitutes a steroid sex hormone that diffuses through the cell membrane due to its hydrophobic nature, enabling extracellular signals to trigger intracellular responses. Traditionally, E2 modulates gene expression by activated ERs binding directly to EREs in gene promoters. Stimulation of cell signaling by E2 could be mediated via homo‐ or heterodimerization of ERα and/or ERβ (Fig. 3A) [16]. However, ERs can also regulate estrogen‐responsive genes lacking EREs through ERE‐independent genomic actions, interacting with DNA transcription factors like c‐Jun, c‐Fos, Sp1, and AP1. Notably, ERα and ERβ exert indirect effects on transcription through protein–protein interactions, stimulating gene expression. Moreover, ERs exhibit rapid non‐genomic interactions, either ligand‐dependent or ligand‐independent (Fig. 3B). Upon E2 binding, ERs are relocated near the cell membrane, activating growth factor receptors (GFRs) and Ca^2+^ channels. Ligand‐independent actions involve the phosphorylation of GFRs, subsequent activation of protein kinase signaling pathways, ER phosphorylation, translocation to the nucleus, and binding to EREs for transcriptional regulation (Fig. 3B). Both genomic and non‐genomic ER actions involve the activation of protein kinase cascades, such as mitogen‐activated protein kinase (MAPK) and phosphoinositide 3‐kinase (PI3K) pathways, as well as signaling molecules such as NO and cAMP production, affecting the expression of various proteins and target genes. These genomic and non‐genomic effects of ERs often intersect, influencing cancer cell functions like proliferation, differentiation, and apoptosis, thus impacting tumor progression (Fig. 3) [17].

## ERs‐matrix interplay affects epigenetic mechanisms within breast cancer cells

Both ERα and ERβ undergo intricate epigenetic regulation that significantly impacts the development and progression of the disease. Epigenetic mechanisms, such as DNA methylation, histone remodeling, and microRNAs (miRNAs) regulation, have been identified as key factors in the epigenetic control of breast cancer, particularly TNBC [18, 19, 20]. These epigenetic signatures are associated with the regulation of various genes and pathways, including LncRNA, HIF‐2α, eEF2K, LIN9/NEK2, IMP3, LISCH7/TGF‐β1, GD3s, and KLK12 [18]. Specific enzymes such as DNA methyltransferases and histone deacetylases play a pivotal role in the formation and maintenance of these epigenetic changes [21].

miRNAs constitute a family of epigenome components participating in cancer development and progression, controlling several processes and modulating ECM molecules expression. Particularly in breast cancer, depending on the functions of mRNA‐target, miRNAs may function either as oncogenes or as tumor suppressors, thus regulating various functional cell properties crucial for breast cancer progression (i.e., proliferation, migration, invasion, apoptosis, metastasis, and chemoresistance) [22]. The intricate communication between different types of cells within the TME has a crucial role in breast cancer progression. Notably, miRNAs serve as mediators of intercellular communication, mostly through their incorporation in extracellular vesicles (EVs). Being carried in EVs, miRNAs contribute to cellular reprogramming, thus promoting various processes such as proliferation, angiogenesis, and epithelial‐to‐mesenchymal transition (EMT) [23]. The role of miRNAs in breast cancer has garnered significant interest, as studies indicate their involvement in regulating ER status, as well as matrix‐guided processes, thus leading to EMT, invasion, and metastasis. Further investigation into the interplay between miRNAs/ERs/ECM and the molecular mechanisms driving breast cancer progression is defined as crucial.

Our group has recently revealed the functional role of ERs to mediate the expression profiles of specific miRNAs associated with breast cancer progression, including miR‐10b, miR‐145, miR‐200b, and let‐7d. Specifically, ERβ controls the expression of miR‐10b and miR‐145 and in turn these miRNAs directly modulate the aggressive functional properties, matrix remodeling, and EMT process in ERβ‐suppressed TNBC [24]. In another study, we have revealed that miR‐200b acts as a suppressor of EMT and aggressiveness in mammary cancer cells, while also influencing matrix composition [25]. Importantly, these effects are found to be dependent on the presence of ER and specific signaling mechanisms. These findings shed light on the intricate regulatory networks involved in mammary cancer progression and highlight potential therapeutic targets hormone‐dependent and hormone‐independent breast cancer [24].

## ERα isoforms control ECM signaling and actions—vice versa effects

Estrogen‐dependent signaling is intimately associated with hormone‐dependent breast cancer subtypes, while ERα is regarded as one of the most important players in breast cancer progression, serving not only as a prognostic biomarker, but also as a predictor of responsiveness to endocrine therapies [2, 14]. We have previously shown that ERα controls breast cancer cell behavior through ECM composition, EMT process, and signaling [26, 27].

**Fig. 4:**
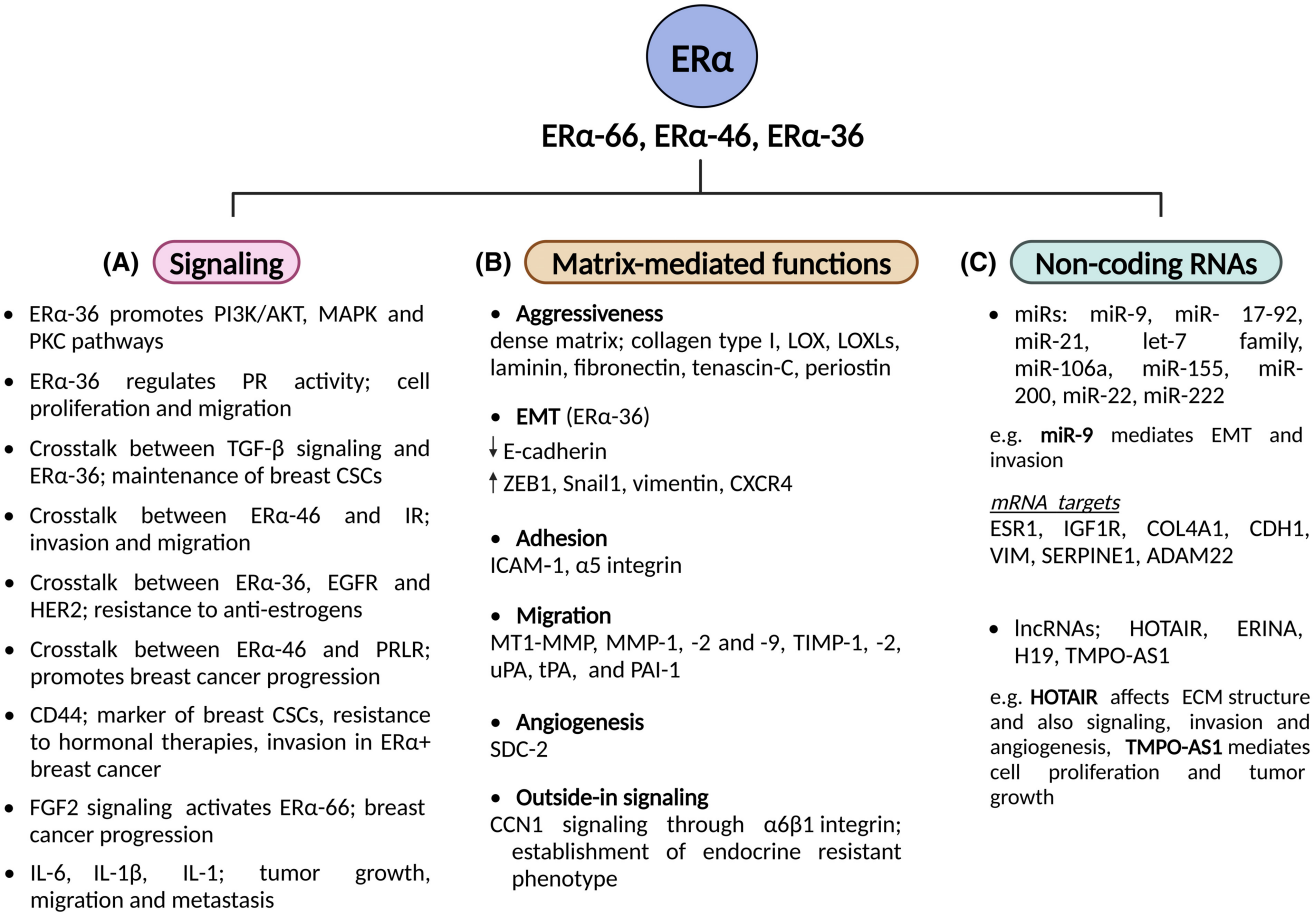
The roles of the main ERα isoforms (ERα‐66, ‐46, ‐36, ‐30) in signaling and breast cancer cell functions modulated by ECM regulators (limited data are available for ERα‐30). ERα isoforms play crucial roles in breast cancer cell signaling and functional properties through interactions with GFRs and other receptors, as well as through the effects of various cytokines. This crosstalk activates downstream signaling pathways, promoting breast cancer cell proliferation, invasion, migration, metastasis, and resistance to anti‐estrogen therapies (A). Different ERα isoforms significantly influence the architecture and composition of the ECM, thereby regulating the aggressive phenotype of breast cancer cells. ECM remodeling affects the EMT process, cell adhesion, migration, angiogenesis, and outside‐in signaling, contributing to the establishment of TME and the consequent progression of breast cancer (B). Estrogen signaling further modulates the expression of non‐coding RNA molecules, which reciprocally interact with ERα isoforms to influence their activity and signaling. Specific miRNAs and lncRNAs (HOTAIR, TMPO‐AS1), regulate the expression of ERα and ECM components. These non‐coding RNAs play distinct roles in various stages of breast cancer development and progression (C). Readers may refer to the text for the corresponding references. Created with BioRender.com. ADAM22, disintegrin and metalloproteinase domain‐containing protein 22; AKT, protein kinase B; CCN1, cellular communication network factor 1; CD44, cluster of differentiation 44; CDH1, E‐cadherin; COL4A1, collagen type IV alpha 1 chain; CSCs, cancer stem cells; CXCR4, CXC motif chemokine receptor 4; ECM, extracellular matrix; EGFR, epidermal growth factor receptor; EMT, epithelial‐to‐mesenchymal transition; ERINA, estrogen‐inducible lncRNA; ERα, estrogen receptor alpha; ESR1, estrogen receptor alpha gene; FGF2, fibroblast growth factor 2; GFRs, growth factor receptors; HER2, human epidermal growth factor receptor 2; HOTAIR, HOX transcript antisense RNA; ICAM‐1, intracellular adhesion molecule 1; IGF1R, insulin‐like growth factor 1 receptor; IL, interleukin; IR, insulin receptor; lncRNAs, long non‐coding RNAs; LOX, lysyl oxidases; LOXLs, LOX‐like isoenzymes; MAPK, mitogen‐activated protein kinase; miR, microRNAs; MMP, matrix metalloproteinase; PAI‐1, plasminogen activator inhibitor 1; PI3K, phosphoinositide 3‐kinase; PKC, protein kinase C; PR, progesterone receptor; PRLR, prolactin receptor; SDC, syndecan; SERPINE1, serpin family E member 1; Snail1, snail family transcriptional repressor 1; TGF‐β, transforming growth factor beta; TIMP, tissue inhibitor of metalloproteinase; TME, tumor microenvironment; TMPO‐AS1, thymopoietin antisense transcript 1; tPA, tissue‐type plasminogen activator; uPA, urokinase‐type plasminogen activator; VIM, vimentin; ZEB1, zinc finger E‐box binding homeobox 1.

Recent attention has been drawn to an ERα isoform located both in the cytoplasm and the plasma membrane, ERα‐36, due to its involvement in resistance to anti‐estrogen therapies, which correlates with unfavorable patient outcomes [14, 28, 29]. In particular, ERα‐36 initiates rapid, non‐genomic signaling pathways that activate the PI3K/AKT, MAPK, and PKC pathways, which further induce the expression of cell cycle genes, such as cyclin D1 and cyclin‐dependent kinases, thereby promoting cell proliferation and enhancing the metastatic potential of cancer cells (Fig. 4) [12, 30]. Furthermore, the activation of the PKC pathway by E2 through ERα‐36 leads to the suppression of JNK activity, thereby contributing to the induction of anti‐apoptotic mechanisms and cancer progression [10].

Growth factors (GFs) and cytokines emerge as key players in cancer development and the initiation of metastasis, while crosstalk of ERα with GFRs and other receptors leads to further downstream signaling pathways [31, 32]. Notably, TGF‐β signaling is closely connected to ERα‐36 signaling, while this interaction has been shown to maintain breast cancer stemness and thus promoting breast cancer progression (Fig. 4) [33, 34]. Furthermore, the interplay between ERα‐46 and insulin receptor in patient‐derived immortalized cells (BCAHC‐1) induces the expression of interleukin 11, a cytokine that regulates the expression of ECM molecules, such as ICAM‐1 and α5 integrin, mediating the invasion and migration of breast cancer‐associated fibroblasts (CAFs) and the consequent metastasis of breast cancer to the lungs (Fig. 4) [8]. Moreover, estrogen's non‐genomic actions are facilitated by the dynamic crosstalk with GFRs, such as epidermal growth factor receptor (EGFR) and insulin‐like growth factor 1 receptor (IGF‐IR) [35]. The coordinated actions of ERs with EGFR and IGF‐IR affect ECM composition (i.e., syndecan expression), Erk1/2‐dependent signaling, and crucial breast cancer cell properties, including migration and adhesion to fibronectin [36, 37].

For instance, the crosstalk among signaling pathways activated by ERα‐36, EGFR, and HER2 has appeared to be significantly involved in the development of resistance to anti‐estrogens [10]. ERα‐36 has also been identified as a regulator of progesterone receptor (PR) activity in breast cancer, exerting control over both signaling and transcriptional processes associated with progesterone, resulting in increased cell proliferation and migration (Fig. 4) [38].

The architecture and composition of the ECM 3D network significantly impacts on the development of breast cancer, regulating various aspects of its progression and the metastatic potential of cancer cells [39]. More specifically, ERα‐36 signaling, interacting with STAT3, has been shown to mediate the upregulation of MMP‐2 and MMP‐9 expression, leading to ECM remodeling and, consequently, facilitating breast cancer cell invasion and migration (Fig. 4) [29]. Furthermore, E2 affects via ERα‐66 the expression of MT1‐MMP, MMP‐1, ‐2 and ‐9, and their endogenous inhibitors (TIMPs), but also the expression of components of the plasminogen activation system, including urokinase‐type plasminogen activator (uPA), tissue‐type plasminogen activator (tPA) and plasminogen activator inhibitor 1 (PAI‐1) (Fig. 4) [26]. In this manner, the ECM remodeling, which is characterized by alterations in the content and the activity of matrix components, results in the establishment of the breast TME [40].

Breast cancer progression is characterized by changes in other structural matrix components, as well as their aberrant deposition in the pericellular microenvironment [41]. For instance, ERα‐66 signaling has been associated with the upregulation of *COL1A1* and enzymes, such as lysyl oxidases (LOX) and LOX‐like isoenzymes (LOXLs), that all mediate matrix stiffening through collagen crosslinking [42, 43]. Several matricellular proteins, including fibronectin, tenascin‐C, and periostin, have also been upregulated in breast TME by E2 signaling (Fig. 4) [43]. Moreover, E2/ERα‐66 and its crosstalk with EGFR signaling pathways induces the upregulation of syndecan‐2, a transmembrane heparan sulfate proteoglycan (HSPG) that is mainly involved in the angiogenesis and the EMT process in ERα+ breast cancer cells (Fig. 4) [2, 44]. Together, these changes affect matrix turnover and result in cancer cell invasion, ultimately leading to the establishment of a PMN and, finally, to metastasis [43].

Epithelial‐to‐mesenchymal transition stands as a critical process in cancer progression and metastasis, endowing cancer cells with increased invasive potential, migratory capacity, and stemness characteristics [45]. ERα‐66 has been mentioned to repress EMT‐related transcription factors and other associated miRNAs, while promoting E‐cadherin expression [2]. From the other hand, ERα‐36 signaling has been shown to facilitate the transition of ERα+ breast cancer cells from an epithelial to a mesenchymal state, by activating certain EMT‐associated molecules, including transcription factors such as ZEB1, Snail1, vimentin, and CXCR4, while simultaneously downregulating the expression of E‐cadherin (Fig. 4) [10, 46].

Estrogen signaling has also been demonstrated to regulate the expression of genes that result in non‐coding transcripts, such as miRNAs and long non‐coding RNAs (lncRNAs) [47]. For instance, miR‐9, miR‐17‐92, miR‐21, let‐7 family, miR‐106a, miR‐155 and miR‐200 family, among others, are upregulated by E2/ERα signaling during breast cancer. miR‐9, for example, is associated with the downregulation of E‐cadherin and thus the promotion of EMT, affecting matrix invasiveness of breast cancer cells [48, 49]. Τhe relationship between miRNAs and ERα activity is bidirectional, as many miRNAs in turn play a key role in the regulation of E2/ERα signaling. Indeed, miRNAs, such as miR‐9, miR‐221, and miR‐222, that directly suppress *ESR1* expression, are correlated with the progression of ERα+ breast cancer subtypes. Besides ERα, other direct mRNA targets of miRNAs families are ECM‐related molecules, such as IGF‐IR, COL4A1, vimentin, SERPINE1, and ADAM22 mRNAs, all of them associated with breast cancer progression (Fig. 4) [48].

Notably, lncRNAs constitute a family of RNA molecules that participate in epigenetic regulation and gene expression. E2/ERα signaling results in the expression of different oncogenic lncRNAs, such as H19, estrogen‐inducible lncRNA (ERINA), HOX transcript antisense RNA (HOTAIR) etc. [47]. Both H19 and ERINA are expressed mostly in ERα+ breast cancer subtypes, promoting cell proliferation [47, 50]. HOTAIR has gained a lot of attention, because of its role in the development and progression of different types of cancers [51]. Interestingly, this lncRNA has an important function in ECM structure and signaling in breast cancer [52]. In addition, it enhances breast cancer cell invasiveness, by upregulating chondroitin sulfotransferase and, thus, mediating chondroitin sulfation [52, 53]. HOTAIR promotes the EMT and also induces the transcription of vascular endothelial growth factor (VEGF), facilitating in this way the angiogenesis and breast cancer progression [54]. Another important lncRNA, TMPO‐AS1, positively regulates the expression of ERα through direct binding to ERα mRNA, mediating cell proliferation and tumor growth in ERα+ breast cancer cells (Fig. 4) [47].

It is worth noticing that certain matrix components have been shown to affect estrogen signaling via ERα, thus promoting breast cancer progression [55]. Notably, cellular communication network factor 1 (CCN1) signaling, through α6β1 integrin, promotes an endocrine‐resistant phenotype, potentially through the direct binding of CCN1 to ERα, thus regulating transcriptional events in ERα+ breast cancer cells (Fig. 4) [56]. Moreover, CD44, a matrix glycoprotein and one of the major hyaluronan receptors, serves as an important marker for breast cancer stem cells (CSCs) and has been correlated with resistance against hormonal therapies and enhanced invasive potential in ERα+ breast cancer (Fig. 4) [57].

Regarding matrix structural molecules, the collagen family, which represents an abundant component of the ECM, contributes both mechanical strength and signaling cues to the cells [5, 58]. Collagen accumulation and collagen crosslinking are the major causes of ECM stiffness in breast cancer [59]. Significantly, studies have revealed that a collagen I‐rich ECM can modify hormonal cues, thereby promoting ERα+ breast cancer, by increasing invasion and pulmonary metastasis [60]. Importantly, stiff‐collagen ECM is known to enhance tumor growth in response to E2 and prolactin, which means that a dense matrix mediates ERα‐46 and prolactin receptor (PRLR) crosstalk to promote breast cancer progression (Fig. 4) [61]. Breast cancer cells have shown a statistically significant *in vitro* upregulation of HOTAIR, when growing in substrates enriched in laminin, suggesting the relation between this lncRNA and ECM composition (Fig. 4) [52]. These data indicate that matrix composition can alter hormonal signals, promoting aggressive behavior in ERα+ breast cancer subtypes, thereby offering mechanistic understanding into their metastatic potential [60].

Paracrine signaling within TME likely serves as the primary mechanism by which stromal cells influence cancer cell behavior, involving the release of various molecules such as GFs and inflammatory cytokines, many of which have been implicated in driving breast cancer progression [59]. The interaction of these signaling molecules with the ECM components allows their storage in a readily available form, rapidly accessed by the cells [62, 63]. Notably, the regulation of GFs, cytokines, and chemokines signaling can occur at the cell surface level, among others, through the action of HSPGs, such as syndecans [64, 65]. Downstream phosphorylation and activation of ERα, prompted by EGF, is believed to induce the proliferation of breast cancer cells, by triggering a unique chromatin‐binding pattern in cooperation with other transcription factor complexes [66]. Moreover, the ECM has been shown to participate in the FGF2 signaling pathway, which activates ERα‐66 and leads to the activation of ER‐responsive genes that facilitate breast cancer progression (Fig. 4) [55]. Finally, IL‐6 and IL‐1β are also associated with increased tumor growth and metastasis in ERα+ breast cancer patients (Fig. 4) [66]. It is therefore plausible to suggest that abnormal ECM composition could influence the activation of GFs and cytokine receptors and downstream pathways that intersect with ERα signaling, altering target gene expression.

## ERβ functional isoforms in ECM‐mediated responses

Despite the thorough investigation into the functional properties of ERα in breast cancer progression, the role of ERβ is less clearly defined, primarily because of the presence of multiple alternatively spliced variants. ERβ can form homodimers, as well as heterodimers with ERα, thereby modulating the activity of ERα [2]. In fact, ERα/ERβ heterodimers are less effective at transactivating target genes compared to ERα homodimers, suggesting that ERβ inhibits the transcriptional activity of ERα [67]. However, breast cancer patients undergoing systemic treatment demonstrate decreased disease‐free survival when their tumors show elevated expression of the *ESR2* (Fig. 1C) [2].

**Fig. 5:**
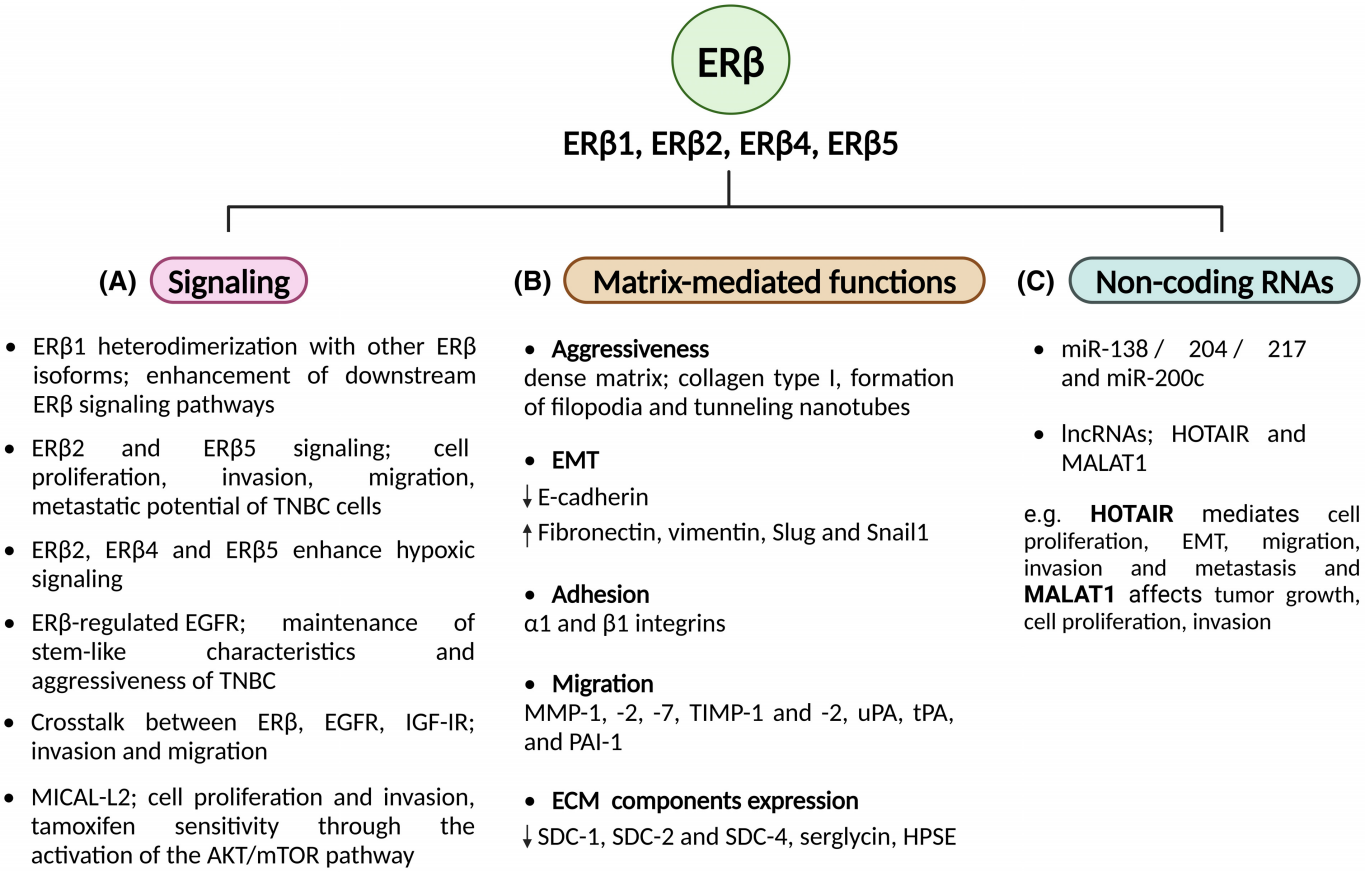
The roles of the main ERβ isoforms (ERβ1–6) in signaling and breast cancer cell functions modulated by ECM regulators (limited data are available for ERβ3 and ERβ6 isoforms). Specific ERβ isoforms play crucial roles in breast cancer cell signaling and functional properties through interactions with GFRs or the induction of other molecules (MICAL‐L2). This crosstalk activates downstream signaling pathways, promoting breast cancer cell proliferation, invasion, migration, metastasis, and resistance to anti‐estrogen therapies (A). Different ERβ isoforms significantly influence the architecture of the ECM and the expression of major matrix molecules, thereby regulating the aggressive phenotype of breast cancer cells. ECM remodeling affects the EMT process, cell adhesion, and migration, contributing to the establishment of TME and the consequent progression of breast cancer (B). Estrogen signaling modulates the expression of some non‐coding RNA molecules, which reciprocally interact with ERβ to influence its activity and signaling. Specific miRNAs (limited data available) and lncRNAs (HOTAIR, MALAT1) regulate the expression of ERβ and ECM components, thus influencing various stages of breast cancer development and progression (C). Readers may refer to the text for the corresponding references. Created with BioRender.com. AKT, protein kinase B; ECM, extracellular matrix; EGFR, epidermal growth factor receptor; EMT, epithelial‐to‐mesenchymal transition; ERβ, estrogen receptor beta; GFRs, growth factor receptors; HOTAIR, HOX transcript antisense RNA; HPSE, heparanase; IGF‐IR, insulin‐like growth factor 1 receptor; lncRNAs, long non‐coding RNAs; MALAT1, metastasis‐associated lung adenocarcinoma transcript 1; MICAL‐L2, molecules interacting with CasL (MICAL)‐like protein 2; miR, microRNAs; MMP, matrix metalloproteinase; mTOR, mammalian target of rapamycin; PAI‐1, plasminogen activator inhibitor 1; SDC, syndecan; Slug, zinc finger transcription factor; Snail1, snail family transcriptional repressor 1; TIMP, tissue inhibitor of metalloproteinase; TNBC, triple‐negative breast cancer; tPA, tissue‐type plasminogen activator; uPA, urokinase‐type plasminogen activator.

Typically, the full‐length ERβ1 is the only isoform that has full ligand‐binding functionality. The functions of ERβ isoforms may diverge given differences in their three‐dimensional structures and abilities to bind to ligands and other molecules, exerting distinct roles in breast cancer development [15, 68]. ERβ1 isoform can usually form heterodimers with other isoforms, thus enhancing the downstream ERβ signaling pathways (Fig. 5) [69]. ERβ2 and ERβ5 are the predominant isoforms concerning breast cancer progression, as their overexpression has been shown to induce functional properties of the breast cancer cells (Fig. 5). For instance, they function in a ligand‐independent way that increases the levels of survival, thus mediating cancer cell proliferation, invasion, and migration, enhancing the metastatic potential of TNBC cells [70]. High ERβ2 mRNA and protein levels have been reported to be associated with worse outcomes in ERα‐ breast cancer, as they have been associated with early‐tumor relapse [68, 71]. Additionally, ERβ2, ERβ4, and ERβ5 isoforms have been shown to enhance hypoxic signaling, thus being correlated with TNBC aggressiveness (Fig. 5) [68].

Additionally, ERβ plays a crucial role in ECM composition during breast cancer progression. Our research group has shown that the establishment of an MDA‐MB‐231 breast cancer cell line with stable suppression of ERβ can influence the expression of ECM molecules. The mature antisense sequences developed to suppress *ESR2* target the 3′ untranslated region (UTR), non‐coding, and open reading frame (ORF) sites, which are common among ERβ variants. Interestingly, *ESR2* suppression attenuates EMT process as it promotes the expression of E‐cadherin and decreases the expression of mesenchymal markers, such as fibronectin, vimentin, Slug, and Snail1, it boosts cell morphology reprogramming, and inhibits of TNBC cells functional properties (Fig. 5). The suppression of ERβ both in mRNA and protein levels, results in a universal matrix remodeling and signaling that has been depicted in the total inhibition of tumor formation *in vivo* [72, 73]. However, contrasting results on mesenchymal phenotype and invasive capacity of breast cancer cells have been acquired by ERβ1 signaling [70].

ECM remodeling and consequent breast cancer cell invasion have been further evaluated by the suppression of ERβ signaling. Specifically, the absence of ERβ leads to the downregulation of matrix enzymes, such as MMP‐1, ‐2, and ‐7, and their endogenous inhibitors, TIMP1 and TIMP2. Additionally, ERβ suppression affects plasminogen activation factors, including uPA, tPA, and PAI‐1 (Fig. 5). This means that ERβ plays a key role in matrix remodeling, thus driving processes such as breast cancer cell invasion and subsequent migration. Furthermore, the absence of ERβ in TNBC cells leads to the upregulation of some critical ECM effectors, such as the cell membrane syndecans (SDC‐1, SDC‐2, and SDC‐4), serglycin and heparanase (HPSE) (Fig. 5) [45]. Moreover, ERβ seems to upregulate the expression of integrins α1 and β1, consequently modifying adhesion (Fig. 5) [74]. MICAL‐L2, a protein that regulates the transport of adhesion molecules and promotes cell–cell adhesion in epithelial cells, also promotes the malignant progression in an ERβ‐dependent manner, mediating cell proliferation and invasion and further inhibiting tamoxifen sensitivity on ER+ breast cancer cells, through the activation of the AKT/mTOR pathway (Fig. 5) [75].

The interaction between ERβ and GFRs activates additional downstream signaling pathways, leading to breast cancer progression. Of note, the ERβ/EGFR/IGF‐IR crosstalk and ERβ/JAK/STAT signaling pathway significantly influence critical breast cancer cell functional properties, such as invasion and migration [72, 76]. Kyriakopoulou *et al*. [76, 77] revealed that ERβ modulation of EGFR might enhance the aggressiveness and stem‐like characteristics of TNBC cells (Fig. 5).

As in the case of ERα, the presence of collagen type I and especially the dense matrix derived from increased collagen I concentration and crosslinking, preserves the aggressive phenotype of MDA‐MB‐231 cells and the consequent formation of long filopodia and tunneling nanotubes (Fig. 5). These structural characteristics of the breast cancer cells result in communication with the ECM and eventually lead to migration and the formation of a PMN [76]. In particular, a correlation between ERβ2 expression and breast density was found following collagen staining [78].

Regarding the epigenetic regulation of ERβ in breast cancer progression, HOTAIR lncRNA crosstalk with ERβ has been shown to promote functional cell properties, including proliferation, EMT, migration, invasion, and metastasis (Fig. 5). Furthermore, it has been found to reduce apoptosis and drug sensibility in breast cancer cells [79]. The correlation between HOTAIR and ERβ, along with subsequent downstream miRNA molecules, such as miR‐138, miR‐204, miR‐217 complex and miR‐200c, in breast cancer, remains to be investigated. Until now, HOTAIR activity has been found to facilitate the metastatic potential of cancer cells, by regulating the expression of VEGF, MMP‐9, and vimentin [80]. In addition, metastasis‐associated lung adenocarcinoma transcript 1 (MALAT1) crosstalk with ERβ has been further associated with effects on breast cancer cell growth, proliferation, and invasive capacity (Fig. 5) [79].

## Concluding remarks and future perspectives

ERs targeting in breast cancer therapy has profound clinical implications. ERα is well known for its role in driving hormone receptor‐positive breast cancer growth; therefore, chemotherapeutics that target ERα signaling, such as aromatase inhibitors and selective estrogen receptor modulators (SERMs), have much improved the survival rates of ERα+ breast cancer patients. However, resistance to these therapies poses a significant challenge, necessitating the development of novel strategies to overcome or prevent resistance. In contrast, ERβ has emerged as a complex but potentially advantageous target. The differential expression of ERβ isoforms in breast cancer subtypes suggests that it could play a role in moderating the aggressive behavior of certain tumors, including TNBC of high metastatic potential. Targeting ERβ, either alone or in conjunction with ERα therapies, might offer a new therapeutic avenue, particularly for patients who develop resistance to conventional ERα‐targeted treatments. Deep understanding of the roles of ERα/β and the underlying mechanisms of their actions as well as the influence of the interconnected ERs' expression profiles , for instance how one ER type effects the expression of the other, are emerging areas of research that could enhance treatment efficacy advancing personalized approaches. Future directions may also consider diagnostic approaches that investigate the expression levels of ERα and ERβ in circulating cancer cells. Combination therapeutic approaches that address both ERα/β signaling and ECM interactions hold promise for more effective breast cancer management. Research into biomarkers for ER activity and resistance mechanisms will also be crucial in optimizing and personalizing ER‐targeted therapies.

## Conflict of interest

The authors declare no conflict of interest.

## Author contributions

SM, NEK, PI, and ZP contributed to interpretation and review of reported data, writing original draft, writing‐review, and editing. ZP, SM, and NEK contributed to figure conceptual design and preparation. NKK contributed to conceptual design, interpretation, writing‐review, and editing. All the authors have read and approved the final version for publication.
